# Four-Year Outcomes of aXess Arteriovenous Conduit in Hemodialysis Patients: Insights from Two Case Reports of the aXess FIH Study

**DOI:** 10.3390/jcm14248768

**Published:** 2025-12-11

**Authors:** Monika Vitkauskaitė, Laurynas Rimševičius, Rokas Girčius, Martijn A. J. Cox, Marius Miglinas

**Affiliations:** 1 Clinic of Gastroenterology, Nephro-Urology and Surgery, Faculty of Medicine, Institute of Clinical Medicine, Vilnius University, 03101 Vilnius, Lithuania; 2 Faculty of Medicine, Vilnius University, 03101 Vilnius, Lithuania; 3 Center of Radiology and Nuclear Medicine, Santaros Clinics, 08661 Vilnius, Lithuania; 4 Xeltis BV in Eindhoven, 5612 AR Eindhoven, The Netherlands

**Keywords:** kidney replacement therapy, hemodialysis, vascular access, dialysis graft

## Abstract

**Background/Objectives**: Arteriovenous grafts (AVGs) are critical for hemodialysis access in patients with inadequate native vasculature. The Xeltis aXess graft, a novel bioresorbable vascular access conduit, promotes endogenous tissue restoration. While early outcomes have been promising, longer-term data remain limited. This report presents the longest reported, four-year follow-up on two of the first implanted aXess devices. **Case Summaries**: *Case 1:* A 64-year-old woman underwent aXess graft placement on 10 June 2021, between the right brachial artery and vein. She experienced graft thrombosis after 12 months and 18 months, both of which were successfully resolved with thrombectomy, in one instance in combination with drug-coated balloon (DCB) angioplasty. The graft remains functional. *Case 2:* A 76-year-old man received an aXess graft on 11 June 2021, in the left arm. After 6 months, he underwent balloon and DCB angioplasty for graft–vein (G–V) anastomosis stenosis. After 28 months, to resolve multiple pseudoaneurysms, followed by aneurysm resection and AVG reconstruction at month 29, a tunneled catheter was placed to perform dialysis sessions in the meantime. At month 44, graft-venous (G–V) angioplasty with DCB was performed to resolve G–V and axillary vein stenoses diagnosed at month 43. The graft remains in use. **Results**: Both patients retained functional dialysis access after four years, despite requiring multiple interventions for thrombosis, stenosis, and pseudoaneurysms. **Conclusions**: These cases demonstrate that the aXess graft can maintain functionality over four years with appropriate management, although close surveillance and reinterventions may be required.

## 1. Introduction

Chronic kidney disease (CKD) has emerged as one of the leading causes of mortality worldwide, affecting more than 800 million individuals [1]. Recent data indicate that the global median prevalence of CKD treated with maintenance hemodialysis or peritoneal dialysis has risen to 823 per million of the population [2]. Hemodialysis (HD) is the predominant form of kidney replacement therapy, accounting for 89% of all dialysis [3]. HD requires reliable vascular access to the patient’s blood circulation, and patients with central venous catheters (CVCs) incur the greatest health care expenditures [4]. Moreover, catheter-associated bacterial infections and sepsis have been the Achilles’ heel of catheters, alongside thrombosis, fibrin sheath formation, and central vein thrombosis [5]. Since 1966, when Brescia-Cimino revolutionized the delivery of hemodialysis therapy by describing a radiocephalic arteriovenous fistula (AVF), it has been considered the optimal access for hemodialysis [4,6]. However, AVFs require sufficient time to mature, adequate arterial and venous size and flow, and a suitable superficial location to allow two-needle cannulation [6]. The median time from AVF creation to overall maturation has been reported at 125 days, with 61% maturation success rate by 6 months [7].

An arteriovenous graft (AVG) provides vascular access through the interposition of a graft segment between an artery and a vein. They have a shorter maturation period and can be used when no suitable vein is available for fistula creation; therefore, they are considered a valid long-term vascular access option for hemodialysis [6]. AVG infection rates have been reported at 9% per year [8], in comparison to 4% per year for AVF [9].

Data on long-term follow-up with AVF/AVG are inherently limited due to a 17% annual mortality rate associated with patients undergoing hemodialysis [10]. Meta-analyses report 2-year secondary patency of 54% for AVG (ePTFE) [8] and 64% for AVF [11]. Longer-term follow-up is typically to single-center reports with very few patients at risk at 3 years and beyond. Interventions for maintaining/restoring patency are common practice for both modalities, with on average 1.8 interventions per year for AVF compared to 3.3 for AVG [12].

**Figure 1:**
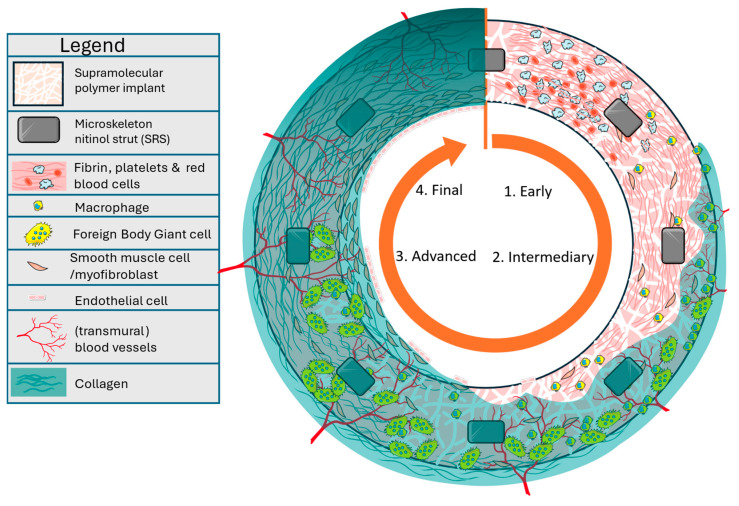
Schematic illustration of the ETR process. Upon implantation, the aXess conduit is sealed by platelets, fibrin, and other blood components. Neotissue and capillaries gradually grow in from the outside, eventually providing a source for the reendothelialization of the lumen. The supramolecular implant microstructure is gradually absorbed by macrophages and giant cells through phagocytosis. The aXess conduit is further supported by an embedded nitinol microskeleton. Image based on Servier Medical Art (https://smart.servier.com), licensed under CC BY 4.0 (https://creativecommons.org/licenses/by/4.0/ (Accessed on 5 December 2025)).

The current study reports long-term four-year follow-ups with two of the first implanted cases of the novel aXess conduit, which has been developed by Xeltis with the intention to combine the best of both approaches. The aXess conduit is a bioabsorbable polymer-based implant that supports shorter maturation periods similar to AVG. Further, the aXess conduit has a microporous structure that allows infiltration by cells and the growth of neotissue, taking over functionality. The underlying supramolecular polymer chemistry [13,14] ensures that the aXess conduit is strong but also very biocompatible and easily absorbed as part of a natural remodeling response called endogenous tissue restoration (ETR) [15,16]. The intended process is schematically illustrated in Figure 1. The porous structure of the aXess conduit is infiltrated with cells directly upon implantation. With time, these cells are followed by collagen-rich neotissue gradually growing in from the outside, accompanied by the ingrowth of new capillaries. Once neotissue and capillaries reach the luminal surface, a neointima is formed and lined with endothelial cells. In parallel, macrophages and giant cells group around remaining polymer fragments which are absorbed through phagocytosis. Importantly, the highly porous microstructure of the aXess conduit provides more infection resistance compared to AVG, because capillary ingrowth enables more effective local delivery of antibiotics and immune cells are capable of infiltrating the aXess pores in response to an infection.

Our center participated in the Xeltis aXess First in Human (FIH) study (NCT04898153) and, after obtaining all regulatory and ethical approvals, implanted the first two study devices in patients who had signed the informed consent and were accepted for study participation by the screening committee. Our patients, prior to the study participation, were discussed within the multidisciplinary team of nephrologists and vascular surgeons. Herein, we present the long-term follow-ups from two illustrative cases from the initial clinical experience with the aXess conduit.

## 2. Case Reports

### 2.1. Case 1

The 64-year-old woman had a history of hypertensive nephropathy diagnosed in November 2020 and hemodialysis was initiated in January 2021. She had no other chronic comorbidities and was treated solely with antihypertensive medication. On 10 June 2021, she underwent the placement of an aXess conduit device by connecting the right brachial artery to the right brachial vein. The surgery was uncomplicated. The patient was cannulated three times a week using the rope-ladder technique (first cannulation on 28 June 2021) and approximately 294 cannulations (two needles in each dialysis session) with needle size 17 G were performed without complications. Follow-up evaluations were scheduled at 1, 3, 6, 12, 18, and 24 months, and yearly thereafter.

**Figure 2:**
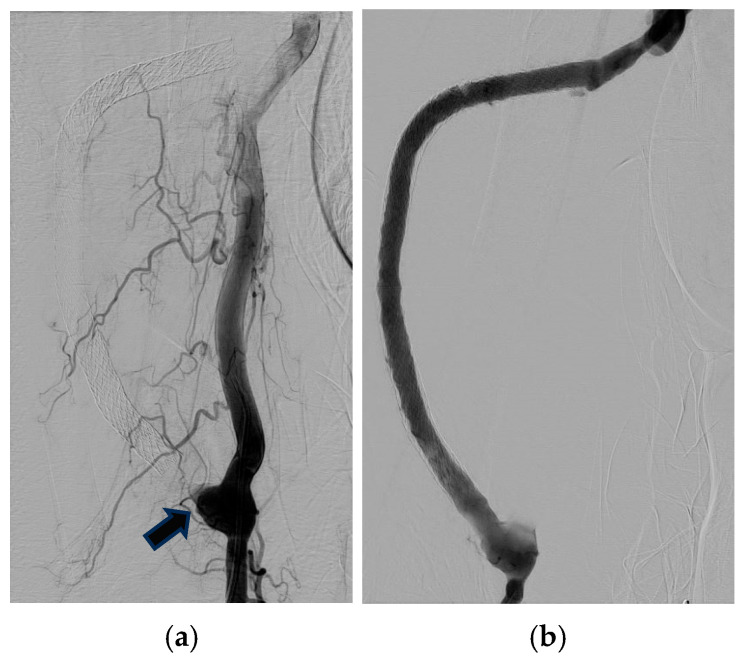
Angiographic views of an arteriovenous graft (AVG) occlusion (arrow) (**a**) and post-intervention result (**b**).

On 6 June 2022, routine ultrasound examination revealed an occlusion near the proximal segment of the graft with a significant decrease in blood flow (1522 mL/min to 114 mL/min), in an AVG originating from the right brachial artery. Angioplasty with a drug-coated balloon (IN.PACT^TM^, Medtronic, MN, USA)) and thrombus aspiration were performed (Figure 2). Following the intervention, the blood flow increased to 1680 mL/min.

Six months after the first thrombectomy (and 572 days after conduit implantation and 178 cannulations with 89 hemodialysis sessions), ultrasound examination was performed due to failed AVG puncture and demonstrated thrombosis of the entire length of the graft, starting from the proximal anastomosis. Thrombectomy and balloon angioplasty were performed at both the arterial and venous anastomotic sites. On both occasions, the patient received antiplatelet therapy with clopidogrel for two months. Re-thrombosis of the AVG was most likely due to recurrent stenosis from neointimal hyperplasia and disturbed flow patterns at the anastomotic sites. Additional contributing factors may include repeated cannulation-related trauma and the patient’s underlying prothrombotic state associated with chronic hemodialysis.

A follow-up ultrasound, performed six months after the second intervention (725 days after implantation), revealed a mild stenosis at the graft–vein anastomosis, a brachial artery aneurysm proximal to the artery–graft anastomosis, and small pseudoaneurysms in the stent-covered segment of the graft. The graft lumen remained patent, without evidence of thrombosis, and therefore no intervention was performed. The access blood flow was 1457 mL/min at the time of the ultrasound.

**Figure 3:**
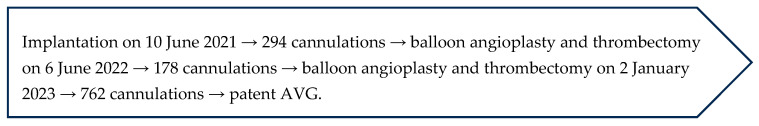
Case 1: Timeline of AVG formation and interventions.

On 13 June 2025, a protocol ultrasound demonstrated persistent stenosis and progressive enlargement of the pseudoaneurysms over time: from 5.6 mm × 5.2 mm × 9.8 mm to 13.6 mm × 11.6 mm × 22.3 mm in the proximal third, and from 3.0 mm × 5.2 mm × 13.6 mm to 12.7 mm × 13.5 mm × 29.1 mm in the distal third of the graft. However, stenosis and pseudoaneurysms had no impact on AVG function (blood flow via AVG—1300 mL/min), and thus no intervention was required. We continued to puncture the conduit with a Kt/V of 1.55 (adequate dialysis) (Figure 3).

In summary, this conduit has been used for cannulation and dialysis for 4 years (>1460 days, 208 weeks, for 3 dialysis sessions per week)—equalizing 1251 cannulations and 625 dialysis sessions. This corresponds to approximately 313 cannulations per patient-year and 156 dialysis sessions per patient-year.

### 2.2. Case 2

Another aXess arteriovenous study device was surgically placed in a 76-year-old man with hypertensive nephropathy on 11 June, 2021, connecting the left brachial artery to the right brachial vein. He had a history of atrial fibrillation and chronic obstructive pulmonary disease, and he had suffered stroke a year prior. His medications included antihypertensives, inhalers, and dual antithrombotic treatment with rivaroxaban and aspirin. At the time of graft placement, he had been on dialysis for two years. His left brachiocephalic arteriovenous fistula was poorly functioning, and he was being dialyzed via permanent central venous catheter. The conduit placement was performed without complications, and the first cannulation was performed on 5 July 2021. The blood flow through the graft was high—2488 mL/min. As in Case 1, the follow-up evaluation schedule was identical, and cannulation was performed using the rope-ladder concept.

Four months after graft implantation (during which 94 cannulations followed by 47 successful dialysis sessions were perfromed), the patient developed a perigraft hematoma. He was evaluated by a vascular surgeon, and it was decided to continue dialysis via a temporary catheter until the hematoma resolved. One month later, a follow-up ultrasound was performed to evaluate the hematoma prior to cannulation. The study revealed a localized support metal strut fracture of the dialysis graft in the middle third, with a likely formation of pseudoaneurysm, as well as a hemodynamically significant graft–vein anastomotic stenosis due to intimal hyperplasia. Localized intimal hyperplasia measured approximately 3 milimeters in thickness, and the peak systolic velocity (Vmax) at the G–V anatomotic stenosis exceeded 500 cm/s. Although the rope-ladder technique was used to distribute cannulation sites, cumulative mechanical stress from repeated puncture and the presence of a perigraft hematoma likely contributed to focal wall weakening. The resulting pseudoaneurysm altered local geometry and may have increased strain on the internal support structure, predisposing it to metal strut fracture. The patient underwent angioplasty with a drug-coated balloon (IN.PACT^TM^, Medtronic, Minneapolis, USA) of the graft–venous anostomosis. AVG cannulation was resumed on 6 December, 2021. Despite intervention, a residual 50% stenosis persisted, which was not further corrected as dialysis adequacy was maintained (Kt/V of 1.47) and the access blood flow remained 1800 mL/min.

Two years later—after 566 uncomplicated cannulations and 283 dialysis sessions—study-related ultrasound showed multiple enlarging graft pseudoaneurysms (PSAs), with the largest located at the arterial–graft anastomosis, with a maximal diameter of 31 mm. At this point, it was decided to implant a permanent venous catheter and continue dialysis via catheter.

**Figure 4:**
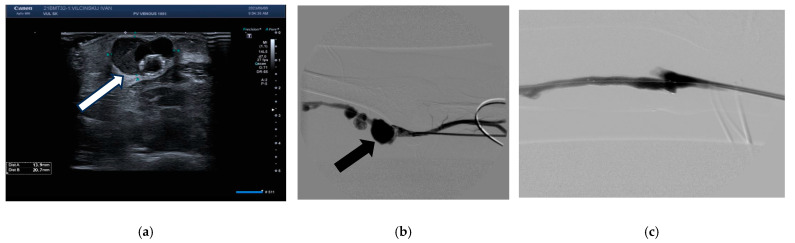
Pseudoaneurysm of arteriovenous graft. Ultrasound (**a**) reveals a well defined pseudoaneurysm cavity (white arrow). Angiography (**b**) demonstrates the pseudoaneurysm with contrast filling (black arrrow). Post-procedure (**c**) confirms exclusion of the pseudoaneurysm and restoration of normal flow through the graft.

However, 51 days after initiation of CVC use, in November 2023, the surgical team decided to excise the pseudoaneurysms, with resection of the sacs and suturing around a drug-coated stent (Figure 4). The surgical procedure was without complications. Following this procedure, the patient alternated dialysis modalities—sometimes via the aXess graft, other times via the CV catheter.

**Figure 5:**
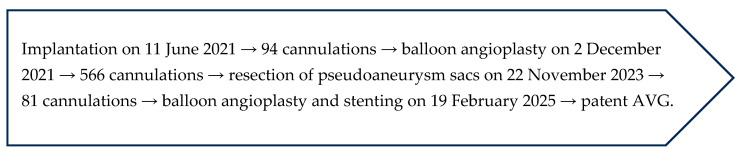
Case 2: Timeline of AVG formation and interventions.

On 19 February 2025 (1350 days after AVG placement), the patient underwent repeat angioplasty and drug-coated stent placement at the graft–venous anostomosis due to restenosis, with the narrowest lumen measuring 2 mm (Figure 5). The procedures were performed without complications. At this point, the patient had undergone approximately 185 cannulations and 93 hemodialysis sessions per patient-year, and the graft remains available for dialysis now beyond 4 years since implantation.

**Figure 6:**
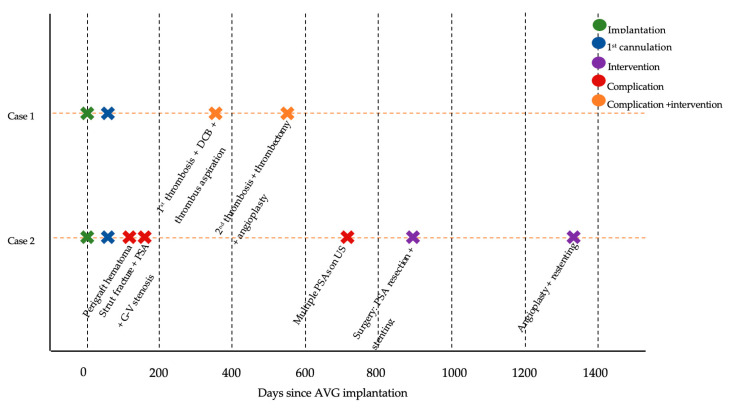
Clinical timeline of key events for both aXess AVG cases.

In both presented cases, the required interventions were technically straightforward and effective, resulting in the prolonged and successful use of conduits. Current guidelines note that maintaining or restoring the patency of AVGs and AVFs generally requires approximately three interventions per year [17]. Remarkably, in these two first implanted devices, the actual number of required interventions was far lower at two interventions in over 4 years for Case 1 and three interventions in over 4 years for Case 2 (Figure 6). No infections were incurred in either case. These findings support that this new technology may offer a potential alternative with improved patient outcomes, reduced intervention burden, and substantial cost savings for the health care system.

## 3. Discussion

The longer life expectancy in the general population, lower dialysis-related mortality, rising prevalence of chronic kidney disease, expansion of eligibility criteria for kidney replacement therapy, and greater access to maintenance dialysis in low- and middle-income countries have contributed to an increase in dialysis patients [18]. Long-term vascular access for hemodialysis patients is crucial but presents distinct challenges, accounting for one third of hospital admissions among patients receiving hemodialysis [3]. Central venous catheters are typically associated with the highest risks of all-cause mortality, fatal infections, and cardiovascular disease [19]. This gave rise to the “Fistula First, Catheter Last” initiative in 2007 [20]. However, as the end-stage kidney disease population expanded, it changed to the “Patient First: ESKD Life-Plan” paradigm in 2019 [17].

The rates of successful AVF and AVG use were 67% and 75% in Europe [3]. Unfortunately, studies have shown that 20–50% of AVFs would fail to mature adequately to vascular access for hemodialysis [21]. Obesity, female sex, and hyperphosphatemia significantly increase the risk of AVF failure [21]. On the other hand, well-functioning AVF also can induce distal ischemia and can produce congestive heart failure [5]. The arteriovenous graft is a catheter-sparing option in patients who have poor fistula maturation and can be produced from various synthetic and biological materials [5]. Conventionally made of polytetrafluoroethylene (PTFE) material, AVG has higher rates of thrombotic occlusion and infections [22].

These two cases reflect the complications that are well described for prosthetic AVGs in general. Prosthetic AVGs are particularly prone to thrombotic failure driven by neointimal hyperplasia at the G–V anastomosis, which results from vascular injury, turbulent flow, compliance mismatch between the graft and native vein, and chronic inflammatory activation—collectively leading to progressive luminal narrowing and recurrent thrombosis [5,8,12,23,24]. Additionally, prosthetic grafts are susceptible to structural degeneration, as repetitive cannulation, external compression, hematoma formation, and material fatigue can induce focal wall stress, increasing the risk of pseudoaneurysm formation and graft rupture, as well as infection due to impaired tissue integrity [5,8,22,24]. Within this known spectrum of AVG complications, Case 1 in our series primarily illustrates the thrombotic failure pathway, whereas Case 2 demonstrates a predominantly structural failure mechanism, where early perigraft hematoma, altered graft geometry, and localized mechanical strain likely promoted pseudoaneurysm formation and—uniquely—supported strut fracture in the aXess conduit.

The aXess conduit is an electrospun absorbable polymer-based microporous implant based on Nobel prize-awarded supramolecular chemistry [14,15,25] that has been designed to overcome the key limitations of both AVF and AVG. In contrast to AVF, aXess does not require a long maturation period prior to cannulation. Also, in contrast to conventional AVG, aXess is based on an absorbable polymer with a porous microstructure that allows cells and tissue to grow in and take over functionality from the gradually absorbing polymer. This is intended to improve long-term patency and reduce the risk of infection associated with permanent polymer implants. Lastly, the proprietary bioresorbable polymer used in aXess is free from intentionally introduced per- and polyfluoroalkyl substances (PFAS).

## 4. Conclusions

The presented, very first ever implanted aXess device cases demonstrate that these innovative grafts can provide durable long-term vascular access, with functional patency maintained for more than four years after placement. Although multiple interventions were required to address usual access-related complications, the grafts remained usable and no infection was observed throughout the 4-year follow-up period. These findings support that the aXess grafts have potential as a safe and reliable option for chronic hemodialysis patients, even in the context of repeated procedures, and may therefore offer benefits for patients and health care system costs. A pivotal European clinical study (NCT05473299) is currently ongoing to substantiate these findings.

## Data Availability

The data supporting the article are available from Monika Vitkauskaitė on reasonable request.
